# Performance Control and Mechanism Analysis of DCLR-Based Composite High-Modulus Asphalt Based on Synergistic Modification Effect

**DOI:** 10.3390/ma19061268

**Published:** 2026-03-23

**Authors:** Bin Xu, Xinjie Yu, Aodong Gao, Guanjun Bu, Kaiji Lu

**Affiliations:** 1 Research Institute of Highway Ministry of Transport, Beijing 100088, China; zlgk_xu@126.com (B.X.); guanjun_bu@163.com (G.B.);; 2 Zhong Lu Gao Ke (Beijing) Road Technology Co., Ltd., Beijing 102600, China

**Keywords:** composite modified asphalt, direct coal liquefaction residue (DCLR), pavement performance, rheological properties

## Abstract

To address the prominent problem of early rutting distress in asphalt pavements under heavy-load traffic in China, this study proposes a composite modifier consisting of direct coal liquefaction residue (DCLR), styrene–butadiene–styrene block copolymer (SBS), and styrene–butadiene rubber (SBR). The preparation process and formula were optimized through single-factor experiments and orthogonal tests. Systematic investigations were conducted on its conventional performance, water damage resistance, aging resistance, fatigue performance, rheological properties, and microscopic mechanism, with comparisons made against base asphalt, single DCLR-modified asphalt, SBS-modified asphalt, and SBS/SBR-modified asphalt. The results indicate that the optimal preparation process for the novel composite high-modulus modified asphalt is as follows: DCLR particle size of 0.3 mm, addition in molten state, shear temperature of 170 °C, shear rate of 5000 r·min^−1^, shear time of 50 min. The optimal formula is 10% DCLR + 3% SBS + 2% SBR + 3% compatibilizer, with the addition sequence of “DCLR → SBS + compatibilizer → SBR”. This asphalt exhibits a softening point of 77.8 ± 2.1 °C, a Brookfield viscosity at 135 °C of 1.928 ± 0.105 Pa·s, and a grading of 5 for adhesion to aggregates; the rutting factor at 64 °C reaches 10.8 ± 0.9 kPa (6.43 times that of the base asphalt), the creep stiffness at −12 °C is 136 ± 12.5 MPa, and the low-temperature limit temperature is −17 °C; the freeze–thaw splitting strength ratio (TSR) is 94.6 ± 1.8%, and both aging resistance and water damage resistance are significantly superior to those of the control group asphalts (*p* < 0.05). The novel composite high-modulus modified asphalt showed improved overall laboratory performance and may be suitable for heavy-load traffic and complex climatic conditions, however, field validation is needed.

## 1. Introduction

Rutting is widely recognized as one of the most critical distresses in asphalt pavements. It is primarily caused by the accumulation of permanent deformation in pavement layers under repeated traffic loading and elevated service temperatures. Excessive rutting can significantly reduce pavement serviceability and ride quality and may also pose safety risks due to water accumulation and reduced vehicle stability. Previous studies have shown that rutting distress is strongly associated with the rheological properties of asphalt binders and the structural stability of asphalt mixtures, particularly under heavy traffic and high-temperature conditions [1]. Consequently, improving the high-temperature performance and rutting resistance of asphalt materials has become an important topic in pavement engineering research [2].

High-modulus modifiers have been applied in newly built asphalt pavements, remarkably improving the high-temperature stability of asphalt mixtures. Their design concept is to increase the modulus of asphalt concrete, thereby reducing plastic deformation of asphalt concrete under vehicle loads, enhancing the high-temperature rutting resistance of pavements, improving the fatigue resistance of asphalt concrete, and extending pavement service life. Although high-modulus modifiers significantly improve the high-temperature performance of asphalt mixtures, they have an adverse impact on their low-temperature performance, which greatly limits their application scope in China [3].

In recent years, domestic researchers have focused on improving the low-temperature crack resistance of high-modulus asphalt pavements. The main research approach is to compound high-modulus modifiers with another type of modifier for asphalt mixture modification, aiming to simultaneously enhance both high-temperature and low-temperature performance [4]. This study focuses on composite high-modulus modifiers composed of direct coal liquefaction residue (DCLR), SBS, and SBR. DCLR modifier is adopted to improve the high-temperature performance and water damage resistance of asphalt, while SBS and SBR modifiers are used to enhance the low-temperature crack resistance and anti-aging performance of asphalt. The ultimate goal is to mitigate early distresses of asphalt pavements and extend their service life, which is consistent with the performance optimization objectives verified in the subsequent conclusions [5].

Due to the remarkable rutting resistance of high-modulus modified asphalt mixtures, numerous researchers have conducted in-depth studies on their service performance. In 2000, China began to introduce PR.S high-modulus modifier from France’s PR Company, which was successfully applied in actual road construction with notable effects and subsequently promoted on a large scale nationwide [6]. Studies have demonstrated that the LuBao brand high-modulus agent achieves the same modification effect as France’s PR.S high-modulus agent, marking the starting point for China to independently solve the problem of rutting lateral flow [7]. Despite the significant improvement in the high-temperature stability of asphalt pavements by adding high-modulus modifiers, their low-temperature performance still requires further enhancement, which lays the foundation for the composite modification scheme proposed in this study [8].

Zou et al. studied the influence of high-modulus modifiers on the pavement performance of asphalt concrete. By combining laboratory tests with the service condition of test road sections after opening to traffic, a gradation design method and construction process suitable for high-modulus asphalt concrete pavements in China were established [9]. Huang et al. investigated the relationship between the modulus of asphalt mixtures and the rutting resistance of asphalt pavements, indicating that the higher the modulus of asphalt mixtures under high-temperature conditions, the better the rutting resistance of asphalt pavements [10].

Studies have revealed that the composition of DCLR modifier is similar to that of Trinidad lake asphalt (TLA), enabling its application in asphalt modification [11,12]. However, research on DCLR is mainly conducted domestically; foreign studies on DCLR modifier are limited to component analysis, with no involvement in modified asphalt research [13]. Zhu et al. compared the performance differences between DCLR-modified asphalt and TLA-modified asphalt, and the results showed that DCLR modifier exhibits a better modification effect on asphalt than TLA, and both DCLR and TLA modify asphalt through physical means [14,15]. Wang et al. studied the adhesion of DCLR-modified asphalt based on the surface free energy theory, investigating the effects of DCLR dosage and asphalt aging on surface free energy and calculating the influence on asphalt adhesion via surface free energy [14,15]. Ma et al. explored the rheological properties of DCLR and compared them with base asphalt; the results indicated that the high-temperature deformation resistance of DCLR is far superior to that of base asphalt, suggesting broad application prospects of DCLR as an asphalt modifier [16]. Izaks et al. studied the performance variation law of asphalt modified by DCLR under different conditions and explored its modification mechanism from a microscopic perspective through scanning electron microscopy (SEM) and Fourier transform infrared spectroscopy (FTIR) tests, providing a theoretical basis for the modification mechanism analysis in the conclusions [17,18].

Although the addition of DCLR effectively enhances the high-temperature performance of asphalt, it exerts a significant adverse impact on its low-temperature performance. Studies have indicated that tetrahydrofuran-insoluble substances in DCLR are the primary factors impairing the low-temperature performance of asphalt [19]. Tetrahydrofuran-soluble (THFS) substances can be extracted from DCLR for asphalt modification, but the purification process is extremely cumbersome. Consequently, some scholars have adopted compound modification with other modifiers to improve the low-temperature performance of asphalt [20]. When compounding with a single modifier, the optimal performance is achieved at an SBS dosage of 3% and a crumb rubber dosage of 15%; excessive dosage beyond these values will result in the degradation of asphalt performance. Notably, compound modification with 2% SBS and 15% crumb rubber can meet the performance requirements for SBS I-D specified in the specifications [21].

Currently, the low-temperature performance of DCLR is the main constraint limiting its application, and the modification mechanism of DCLR has not been systematically investigated. To promote the wide application of DCLR in practical engineering, the primary issue to address is ensuring satisfactory low-temperature performance while maximizing the DCLR dosage [22]. This poor low-temperature performance may stem from the inadequate compatibility between DCLR and asphalt. Therefore, compatibilizers can be added to form a stable dispersion structure of DCLR in asphalt, and other modifiers can be compounded to comprehensively enhance the low-temperature performance of asphalt [23]. Gao et al. studied the effect of coal tar residue (similar to DCLR in composition) on asphalt performance, pointing out that coal-based modifiers can significantly improve the high-temperature modulus of asphalt, but the low-temperature ductility decreases by 30–40%, which is consistent with the defect of single DCLR modification [24]. Their research also found that the compatibility between coal-based modifiers and asphalt can be improved by adding polymer modifiers, but no specific combination scheme of three components was proposed. Ji et al. conducted microscopic analysis on DCLR-modified asphalt by SEM and FTIR, confirming that DCLR is dispersed in asphalt in the form of particles, and its modification mechanism is mainly physical blending [25]. This supports the conclusion of this study that the DCLR/SBS/SBR composite system is mainly physical modification. However, their research only focused on single DCLR modification and did not explore the synergy with polymers [26].

Recently, Xia et al. published a study on coal-based-composite-modified asphalt, pointing out that the combination of coal ash and SBS can improve the high-temperature performance of asphalt, but the low-temperature performance is still insufficient, and it is necessary to add a third component with low-temperature flexibility [27]. This further verifies the necessity of introducing SBR into the DCLR/SBS system in this study, which fills the gap of international research on three-component composite modification of coal-based modifiers. International studies on SBS/SBR dual-component modification are relatively in-depth, which can be compared with the three-component system in this study. Zhi et al. studied the effect of SBS/SBR ratio on asphalt performance, finding that the optimal ratio can improve the fatigue life by 2–3 times, but the high-temperature rutting factor is only 5–7 kPa, which is far lower than the 10.8 kPa of the DCLR/SBS/SBR composite asphalt in this study [28]. This shows that the introduction of DCLR significantly enhances the high-temperature stability of the SBS/SBR system. Ji et al. evaluated the aging resistance of SBS/SBR-modified asphalt through long-term pavement monitoring, pointing out that the residual penetration ratio after PAV aging is about 55–60% [29]. In this study, the residual penetration ratio of DCLR/SBS/SBR composite asphalt after PAV aging reaches 62.2%, and the viscosity aging index is only 0.0457, which is lower than that of SBS/SBR-modified asphalt (0.0482). This indicates that DCLR can improve the aging resistance of the composite system.

In view of the scarce domestic research and application of new composite high-modulus asphalt mixtures based on DCLR, few scholars have explored the decay law of various pavement performances under the combined effects of long-term traffic loads, environmental dry–wet alternation, freeze–thaw cycles, and temperature fatigue [30]. Whether its long-term pavement performance can meet the service requirements of asphalt pavements and achieve the designed service life remains to be verified through scientific experiments and engineering practice [31].

To address the above gap, this study proposes a DCLR–SBS–SBR composite high-modulus asphalt based on a designed rigid–elastic–tough synergy. DCLR forms the high-modulus backbone for rutting resistance, SBS establishes an elastic polymer network to improve recoverable deformation, and SBR provides additional toughness and stress relaxation capacity to offset DCLR-induced low-temperature brittleness. To ensure multiphase stability, a maleic-anhydride-grafted compatibilizer is incorporated to enhance interfacial affinity and dispersion stability, consistent with compatibilization principles reported for polymer-modified asphalt systems. Importantly, the proposed preparation route uses a sequence-controlled blending strategy (“DCLR → SBS + compatibilizer → SBR”) as a morphology-regulating lever to promote SBS swelling/network development while limiting SBR thermal degradation and mitigating phase separation. The novelty therefore lies in establishing a composition–process–morphology–performance linkage for a DCLR-centered high-modulus binder and demonstrating a practical pathway to achieve simultaneous high-temperature reinforcement and specification-compliant low-temperature performance.

## 2. Materials and Test Methods

### 2.1. Raw Materials

#### 2.1.1. Base Asphalt

The SK-90 base asphalt produced by Xiamen Huate was selected for the test. The results are shown in Table 1. 

#### 2.1.2. Direct Coal Liquefaction Residue (DCLR)

The DCLR used in the test was provided by Shaanxi Shenhua Group, Weinan, China. It was in block form and crushed into powder with a crusher before use. The test results of its performance indicators are shown in Table 2.

#### 2.1.3. SBS Modifier

A linear SBS modifier (model: YH-792) was selected, and its performance indicators are shown in Table 3. This modifier has the dual characteristics of plastic and rubber, which can effectively improve the high- and low-temperature performance of asphalt.

#### 2.1.4. SBR Modifier

The SBR modifier produced by Shandong Xianyuan Co., Ltd., Laiyang, China was selected, which is in the form of white powder. Its performance indicators are shown in Table 4.

#### 2.1.5. Compatibilizer and Stabilizer

To improve the compatibility between DCLR, SBS, SBR, and base asphalt, maleic-anhydride-grafted polyethylene (MAH-g-PE) with a grafting rate of 1.5% was selected as the compatibilizer; to improve the storage stability of the composite-modified asphalt, sulfur with a purity of ≥99.5% was selected as the stabilizer.

#### 2.1.6. Aggregates

Basalt aggregates and limestone mineral powder were selected for the test, and the aggregate gradation adopted the AC-13 type. The basic performance of each grade of aggregate and mineral powder is shown in Table 5 and Table 6, respectively, all of which meet the requirements of JTG F40-2004.

### 2.2. Test Methods

#### 2.2.1. Preparation Process of Modified Asphalt

(1) Preparation of DCLR-modified asphalt: Heat the base asphalt to 135 °C, heat the DCLR with a particle size of 0.3 mm to a molten state at 190 °C, add it to the base asphalt in proportion, and shear at 170 °C and 5000 r·min^−1^ for 50 min using a high-speed shear mixer to obtain DCLR-modified asphalt. (2) Preparation of novel composite high-modulus modified asphalt: Based on the preparation process of DCLR-modified asphalt, first add SBS and compatibilizer, shear at 180 °C and 5000 r·min^−1^ for 35 min, then add SBR, reduce the temperature to 170 °C and continue shearing for 30 min, finally add the stabilizer and shear for 10 min. After shearing, cure in an oven at 150 °C for 1 h to obtain the novel composite high-modulus modified asphalt.

#### 2.2.2. Conventional Performance Tests

In accordance with JTG E20-2011 Test Procedures for Asphalt and Asphalt Mixtures in Highway Engineering, the 25 °C penetration (T 0604-2011), 10 °C ductility (T 0605-2011), softening point (ring-and-ball method, T 0606-2011), and 135 °C Brookfield viscosity (rotational viscometer method, T 0620-2000) of the modified asphalt were tested to evaluate its basic pavement performance. All tests were conducted with three parallel replicates; the test results met the precision requirements specified in each substandard (e.g., the allowable difference of softening point between parallel tests is ≤0.5 °C).

#### 2.2.3. Water Damage Resistance Tests

(1) Asphalt–aggregate adhesion test: Adopt the boiling method (T 0616-2011), immerse the aggregate particles coated with modified asphalt in boiling water for 3 min, observe the peeling of the asphalt film, and evaluate the adhesion grade. Three parallel replicates were set for each asphalt type, and the dominant grade of the three replicates was taken as the final adhesion grade. (2) Freeze–thaw splitting test: Prepare Marshall specimens in accordance with T 0729-2000, divide them into a standard group and a freeze–thaw group (freeze at −18 °C for 16 h, keep warm at 60 °C for 24 h), conduct the splitting test, and calculate the freeze–thaw splitting strength ratio (TSR), with TSR ≥ 80% meeting the requirement. Six Marshall specimens were prepared for each group (standard/freeze–thaw), and the average splitting strength of six specimens was used for TSR calculation. (3) Immersion Marshall test: Test the standard Marshall stability and immersion Marshall stability in accordance with T 0709-2011 and calculate the immersion stability ratio (MS_0_). Six parallel specimens were set for each test group, in line with the precision requirements of T 0709-2011 (allowable deviation of Marshall stability ≤ 1.0 kN).

#### 2.2.4. Fatigue Performance Test

A four-point bending fatigue test (T 0739-2011) was adopted. Beam specimens of 380 mm × 63.5 mm × 50 mm were prepared, and the test was conducted under a strain-controlled mode at 25 °C with strain levels of 200 µε, 300 µε, and 400 µε and a loading frequency of 10 Hz. The fatigue life when the specimen fails was recorded. Four parallel beam specimens were set for each strain level and each asphalt type; the fatigue life was expressed as the geometric mean of the four replicates.

#### 2.2.5. Rheological Performance Tests

(1) DSR test: Use an AR2000ex dynamic shear rheometer produced by TA Instruments, USA to conduct temperature sweep tests in accordance with AASHTO T315. The temperature range is 52 °C~82 °C with an interval of 6 °C, and the frequency is 10 rad/s. The complex shear modulus (G*) and phase angle (δ) were tested, and the rutting factor (G*/sinδ) and fatigue factor (G*·sinδ) were calculated. Three parallel asphalt samples were tested for each asphalt type; the test curves of the three replicates were overlapped to confirm repeatability, and the mean value was taken as the final result. (2) MSCR test: In accordance with AASHTO TP70, after RTFOT aging of the asphalt sample, apply constant stresses of 0.1 kPa and 3.2 kPa at 64 °C and 70 °C respectively, conduct 10 creep recovery cycles (loading for 1 s, unloading for 9 s), and test the non-recoverable creep compliance (Jnr) and creep recovery rate (R). Two parallel replicates were set for each temperature-stress condition; the allowable relative deviation of Jnr between replicates is ≤15%. (3) BBR test: Use a BBR rheometer to test the creep stiffness (S) and stiffness change rate (m) of asphalt at −6 °C, −12 °C, and −18 °C in accordance with AASHTO T313 to evaluate the low-temperature cracking resistance. Three parallel asphalt beams were prepared for each temperature; the mean value of S and m was taken as the test result, meeting the precision requirement of AASHTO T313-22.

#### 2.2.6. Aging Resistance Test

The rolling thin film oven test (RTFOT, T 0610-2011) was used to simulate short-term thermo-oxidative aging, and the pressure aging vessel (PAV, T 0631-2014) test was used to simulate long-term thermo-oxidative aging. The conventional performance and DSR indicators of asphalt before and after aging were tested, and the viscosity aging index, residual penetration ratio, and ductility retention rate were calculated. Three parallel asphalt samples were used for RTFOT and PAV aging respectively; the aging indicators were calculated based on the mean value of the post-aging performance tests.

#### 2.2.7. Storage Stability Test

In accordance with T 0661-2011, the modified asphalt was placed in an aluminum tube and stored at a constant temperature of 163 °C for 48 h. The softening points of the asphalt at 1/3 of the top, middle, and bottom of the aluminum tube were measured. The storage stability was evaluated by the difference in softening points between the top and bottom, with a difference of <2.5 °C which meets the requirement. Two parallel aluminum tubes were set for each asphalt type; the average softening point difference of the two replicates was taken as the final result.

#### 2.2.8. Microscopic Analysis

Fourier transform infrared spectroscopy (Nicolet iS50) producted by Thermo Fisher Scientific, Madison, WI, USA was used for infrared spectrum analysis, and the modification mechanism was explored through changes in characteristic functional groups. Two parallel asphalt samples were tested for each asphalt type; the infrared spectra of the two replicates were consistent, and the characteristic peak absorbance was calculated as the mean value.

#### 2.2.9. Statistical Analysis

One-way analysis of variance (one-way ANOVA) was adopted, with “asphalt type” or “experimental factor level” as the independent variables and various performance indicators as the dependent variables. All statistical analyses were performed using SPSS 26.0 software with a significance level of α = 0.05. Before ANOVA, the Shapiro–Wilk test (normality) and Levene’s test (homoscedasticity) were conducted to verify the ANOVA assumptions, and all test data met the normality (*p* > 0.05) and homoscedasticity (*p* > 0.05) requirements (no abnormal values were detected via Grubbs test, α = 0.05). If ANOVA indicated a significant difference among groups (*p* < 0.05), Tukey’s HSD test (α = 0.05) was used for pairwise multiple comparisons to identify the source of significant differences between groups. All experimental tests were set with 2–6 parallel replicates in accordance with JTG E20-2011 [28], and the arithmetic mean of valid replicates was used for statistical analysis.

## 3. Results and Discussion

### 3.1. Optimization of DCLR-Modified Asphalt Preparation Process

#### 3.1.1. Effect of Shear Temperature

**Figure 1:**
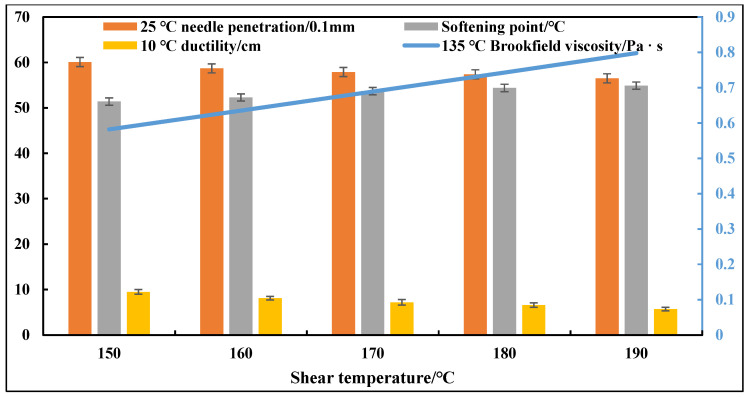
Performance test results of DCLR-modified asphalt at different shear temperatures.

Controlling the DCLR particle size of 0.3 mm, addition in molten state, shear rate of 5000 r·min^−1^, and shear time of 50 min, the effect of shear temperature (150 °C, 160 °C, 170 °C, 180 °C, 190 °C) on the performance of DCLR-modified asphalt was investigated, and the results are shown in Figure 1.

Figure 1 shows that, with the increase in shear temperature, the penetration of DCLR-modified asphalt gradually decreases, the softening point gradually increases, and the 10 °C ductility gradually decreases. To verify the significance of the effect of shear temperature on various performance indicators, a one-way ANOVA was conducted, and the results are shown in Table 7.

Table 7 indicates that shear temperature had an extremely significant effect on 25 °C penetration (F = 32.24, *p* = 0.000), softening point (F = 24.25, *p* = 0.000), 10 °C ductility (F = 24.24, *p* = 0.000), and Brookfield viscosity at 135 °C (Pa·s) (F = 21.89, *p* = 0.000). Multiple comparison results showed that there was no significant difference in penetration between the 170 °C group and the 180 °C and 190 °C groups, but the ductility of the 170 °C group was significantly higher than that of the 180 °C and 190 °C groups (*p* < 0.05), and the softening point had reached an optimal level. In addition, the increase in shear temperature increased the adhesion grade between DCLR and aggregates from 3 to 4. Taking into account the high- and low-temperature performance, adhesion, and construction feasibility comprehensively, the optimal shear temperature was determined to be 170 °C.

#### 3.1.2. Effect of DCLR Particle Size

**Figure 2:**
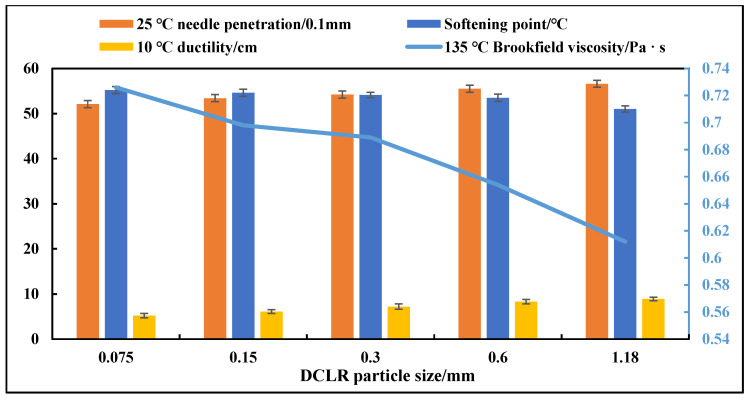
Performance test results of modified asphalt with different DCLR particle sizes.

Controlling the shear temperature of 170 °C, addition in molten state, shear rate of 5000 r·min^−1^, and shear time of 50 min, the effect of DCLR particle size (0.075 mm, 0.15 mm, 0.3 mm, 0.6 mm, 1.18 mm) on the performance of modified asphalt was investigated, and the results are shown in Figure 2.

ANOVA results show (Table 8) that DCLR particle size has an extremely significant effect on the 25 °C penetration (F = 32.24, *p* < 0.001), softening point (F = 24.25, *p* < 0.001), and 10 °C ductility (F = 24.24, *p* < 0.001) of modified asphalt. Multiple comparisons indicate that the softening point of the groups with particle size ≤ 0.3 mm is significantly higher than that of the groups with particle size > 0.3 mm (*p* < 0.05), and the ductility of the 0.3 mm group is significantly higher than that of the 0.075 mm and 0.15 mm groups (*p* < 0.05). In addition, when the particle size is ≤0.3 mm, the adhesion grade between asphalt and aggregates is 4, while it decreases to 3 when the particle size is >0.3 mm. Overall, the optimal DCLR particle size is determined to be 0.3 mm.

#### 3.1.3. Effect of DCLR Addition Method

**Figure 3:**
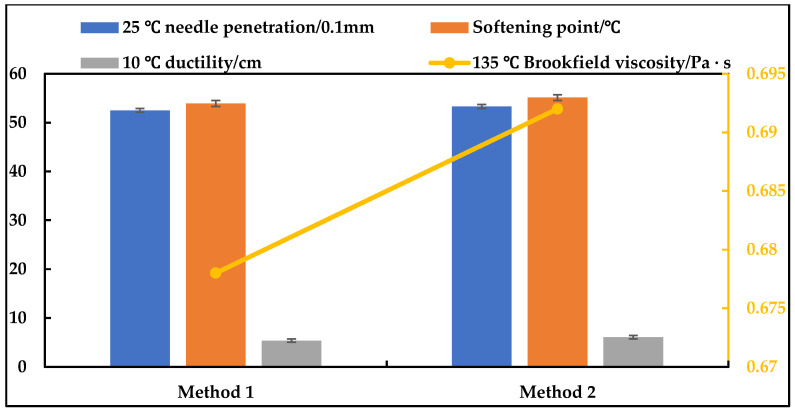
Performance test results of modified asphalt with different DCLR addition methods.

Controlling the shear temperature of 170 °C, DCLR particle size of 0.3 mm, shear rate of 5000 r·min^−1^, and shear time of 50 min, the effect of two addition methods on the performance of modified asphalt was investigated: Method 1: Direct addition of powdered DCLR; Method 2: Addition of DCLR after heating to molten state. The results are shown in Figure 3.

Figure 3 indicates that the DCLR-modified asphalt prepared by Method 2 is superior to Method 1 in terms of softening point, ductility, adhesion grade, and storage stability. This improvement is attributed to the more uniform dispersion of molten DCLR in the asphalt matrix, which reduces particle agglomeration. Accordingly, adding DCLR in the molten state was selected as the preferred procedure.

### 3.2. Optimization of Novel Composite High-Modulus Modified Asphalt Formula

#### 3.2.1. Determination of DCLR Dosage Range

**Figure 4:**
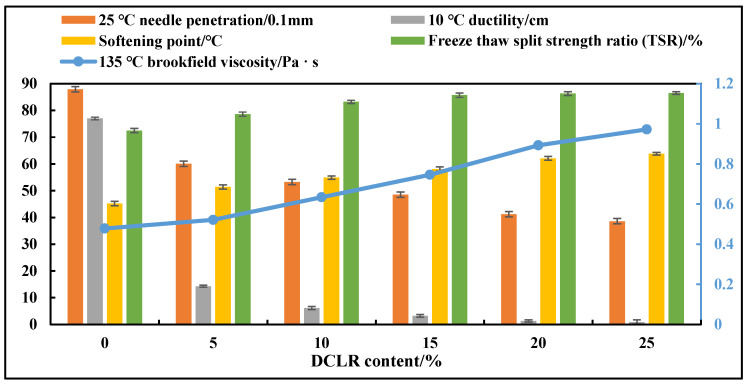
Asphalt performance test results with different DCLR dosages.

Keeping other conditions unchanged, the effect of DCLR dosage (5%, 10%, 15%, 20%, 25%) on the performance of modified asphalt was investigated, and the results are shown in Figure 4.

Figure 4 shows that, with the increase in DCLR dosage, the asphalt penetration gradually decreases, the softening point and viscosity gradually increase, the water damage resistance gradually improves, but the low-temperature ductility decreases significantly. When the DCLR dosage exceeds 15%, the improvement in high-temperature performance tends to slow down, while the low-temperature performance continues to deteriorate. Considering the combined effects of the high- and low-temperature performance, water damage resistance, and cost comprehensively, the DCLR dosage range is determined to be 5%, 10%, and 15%.

#### 3.2.2. Determination of SBS Dosage Range

**Figure 5:**
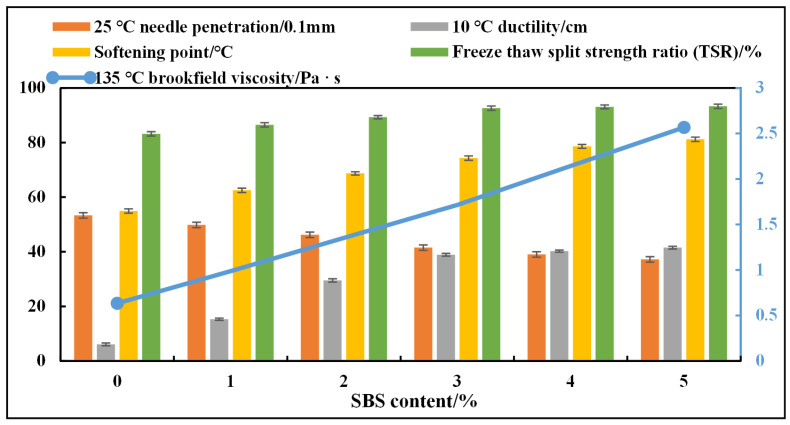
Asphalt performance test results with different SBS dosages.

Fixing the DCLR dosage at 10%, the effect of SBS dosage (1%, 2%, 3%, 4%, 5%) on the performance of DCLR/SBS-composite-modified asphalt was investigated, and the results are shown in Figure 5. It can be seen from Figure 5 that, with the increase in SBS dosage, the penetration of the composite-modified asphalt decreases, and the softening point, viscosity, ductility, and water damage resistance all gradually improve. However, when the SBS dosage exceeds 3%, the performance improvement tends to slow down significantly. Taking into account the modification effect and cost comprehensively, the SBS dosage range is determined to be 1%, 2%, and 3%.

#### 3.2.3. Determination of SBR Dosage Range

**Figure 6:**
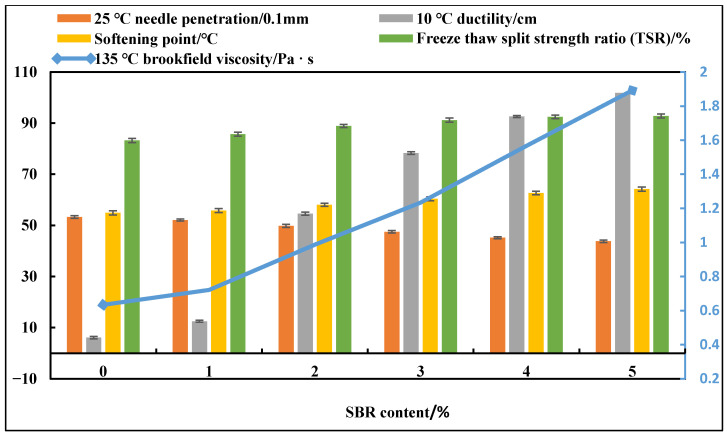
Asphalt performance test results with different SBR dosages.

Fixing the DCLR dosage at 10%, the effect of SBR dosage (1%, 2%, 3%, 4%, 5%) on the performance of DCLR/SBR-composite-modified asphalt was investigated, and the results are shown in Figure 6. It can be seen from Figure 6 that SBR has a significant effect on improving the low-temperature ductility of asphalt. When the SBR dosage increases from 0% to 2%, the 10 °C ductility increases from 6.1 cm to 54.6 cm, an increase of 8.9 times. However, when the SBR dosage exceeds 4%, the performance improvement tends to slow down. The SBR dosage range is determined to be 2%, 3%, and 4%.

#### 3.2.4. Optimization of Modifier Addition Sequence

Three modifier addition sequences were designed to investigate their effects on the performance of the composite-modified asphalt. The modifier dosages were fixed as 10% DCLR, 3% SBS, 2% SBR, and 2% compatibilizer, and the results are shown in Table 9. It can be seen from Table 9 that the composite-modified asphalt prepared by Process 2 is superior to Process 1 and Process 3 in terms of ductility, TSR, and storage stability. In Process 3, the SBR ages due to high-temperature shearing after being added first, resulting in deteriorated low-temperature performance; in Process 1, the modifiers are added simultaneously, making it difficult to achieve sufficient swelling and cross-linking, resulting in slightly inferior performance. Therefore, the optimal addition sequence is determined to be “DCLR → SBS + compatibilizer → SBR”.

#### 3.2.5. Orthogonal Test for Optimal Formula

A four-factor and three-level (L9(3^4^)) orthogonal test (in accordance with GB/T 3715-2008 ≪Orthogonal Test Design Method≫) was adopted, with the factors being DCLR dosage (A: 5%, 10%, 15%), SBS dosage (B: 1%, 2%, 3%), SBR dosage (C: 2%, 3%, 4%), and compatibilizer dosage (D: 1%, 2%, 3%). Each orthogonal test group was set with three parallel replicates; all performance indicators in Table 10 are the arithmetic mean of the three replicates, and the test data met the precision requirements of the corresponding test standards. The test results are shown in Table 10, and the range analysis results are shown in Table 11.

The range analysis (Table 11) was used to determine the primary and secondary order of factors affecting each performance indicator, and the optimal level of each single factor was selected according to the effect trend of factor levels on key performance indicators.

The extreme difference (R) reflects the influence degree of each factor on the performance indicators. DCLR dosage (A) is the primary factor affecting penetration and ductility (R = 7.9, 17.4), SBS dosage (B) is the primary factor affecting softening point and viscosity (R = 6.9, 0.607), and the four factors have little effect on storage stability (R ≤ 0.3). The optimal level of each factor was determined by combining the R value and the actual performance improvement effect.

All performance indicators of the optimal formulation must meet the requirements of JTG F40-2004 Technical Specifications for Construction of Highway Asphalt Pavements (e.g., 10 °C ductility ≥ 10 cm, TSR ≥ 80%, storage stability softening point difference < 2.5 °C): Avoid excessive pursuit of high-temperature performance (e.g., excessive DCLR dosage) leading to severe deterioration of low-temperature ductility, and avoid excessive polymer dosage (SBS/SBR) leading to excessive viscosity and increased engineering cost; on the premise of meeting performance requirements, select the appropriate dosage of modifiers (especially high-cost SBS/SBR) to reduce the preparation cost of modified asphalt; the compatibilizer dosage is determined by the compatibility effect (avoid excessive dosage with no significant performance improvement).

DCLR dosage has an extremely significant effect (*p* < 0.001) on penetration and ductility, SBS dosage has an extremely significant effect (*p* < 0.001) on softening point and viscosity, and the interaction between factors is not significant (*p* > 0.05). The optimal level of each factor was further confirmed based on the significance test results to avoid the selection bias of a single range analysis.

Combined with the above dual criteria and ANOVA statistical results, the optimal formulation of the novel composite high-modulus modified asphalt was determined as: 10% DCLR (A2) + 3% SBS (B3) + 2% SBR (C1) + 3% compatibilizer (D3). This formulation not only corresponds to the optimal level combination of key performance indicators in the range analysis but also meets all the performance requirements of JTG F40-2004, realizing the balance of high/low-temperature performance, aging resistance, storage stability, and engineering economic efficiency.

### 3.3. Performance Evaluation of Novel Composite High-Modulus Modified Asphalt

#### 3.3.1. Conventional Performance

The comparison results of conventional performance between the novel composite high-modulus modified asphalt and the control group asphalts are shown in Table 12. ANOVA results show that there are extremely significant differences in various conventional performance indicators among different asphalt types (*p* < 0.001). Multiple comparisons indicate that the softening point and viscosity of the novel composite high-modulus modified asphalt are significantly higher than those of other asphalts (*p* < 0.05), the penetration is significantly lower than that of other asphalts (*p* < 0.05), and the 10 °C ductility, although lower than that of SBS-modified asphalt and SBS/SBR-modified asphalt, is significantly higher than that of DCLR-modified asphalt and base asphalt (*p* < 0.05), meeting the specification requirements. The adhesion grade to aggregates reaches 5, indicating excellent interface bonding performance.

#### 3.3.2. Water Damage Resistance

**Figure 7:**
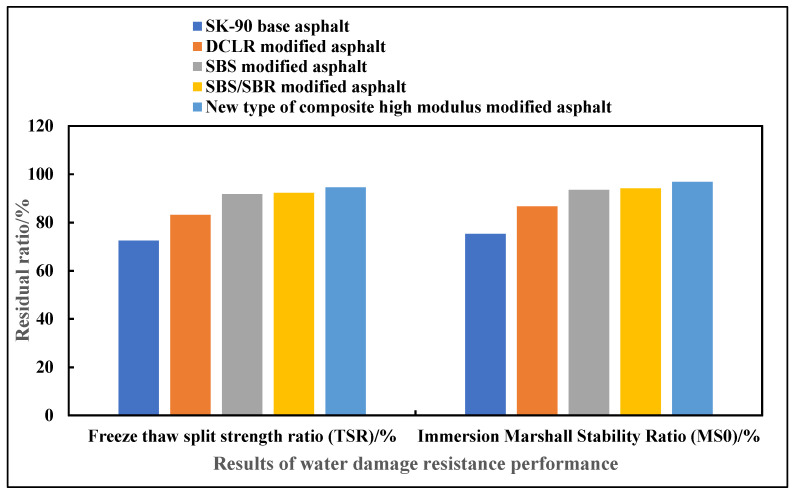
Comparison results of water damage resistance of five types of asphalt.

The comparison results of water damage resistance of five types of asphalt are shown in Figure 7. ANOVA results show that there are extremely significant differences in TSR and MS_0_ among different asphalt types (*p* < 0.001). The TSR of the novel composite high-modulus modified asphalt is 94.6 ± 1.8% (95% CI: [92.3, 96.9]%), and the MS_0_ is 96.8 ± 2.0% (95% CI: [94.1, 99.5]%), which are significantly higher than those of SBS/SBR-modified asphalt (TSR: 92.3 ± 2.1%) and SBS-modified asphalt (TSR: 91.8 ± 2.3%) (*p* < 0.05), far exceeding the specification requirement of 80%, and the water damage resistance is the best.

#### 3.3.3. Fatigue Performance

**Figure 8:**
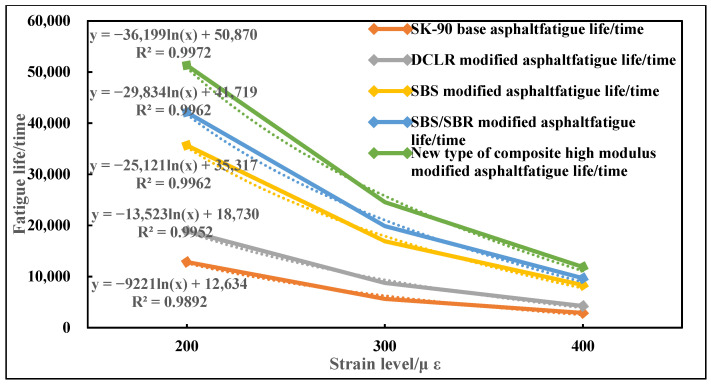
Comparison results of fatigue performance of five types of asphalt mixtures (25 °C). Note: The dotted line is the fitting formula.

The four-point bending fatigue test results of five types of asphalt mixtures are shown in Figure 8. ANOVA results show that there are extremely significant differences in fatigue life among different asphalt types (*p* < 0.001). The fatigue life of the novel composite high-modulus modified asphalt is the longest at all strain levels, reaching 51,320 ± 3860 times (95% CI: [46,580, 56,060] times) at 200 µε, which is 4.0 times that of the base asphalt (12,860 ± 1120 times) with significant statistical difference (*p* < 0.001).

#### 3.3.4. Aging Resistance

The performance indicators of five types of asphalt after RTFOT short-term aging and PAV long-term aging are shown in Table 13 and Table 14, respectively, and the calculation results of aging evaluation indicators are shown in Table 15. It can be seen from Table 15 that the novel composite high-modulus modified asphalt has the smallest viscosity aging index and the largest residual penetration ratio and ductility retention rate after both RTFOT and PAV aging. The ranking of aging resistance is: Novel composite high-modulus modified asphalt > SBS-modified asphalt > SBS/SBR-modified asphalt > DCLR-modified asphalt > SK-90 base asphalt.

The excellent aging resistance of the composite asphalt is due to the stable physical entanglement structure (in FTIR, no new characteristic peaks after aging) and DCLR’s aromatic ring structure inhibiting the oxidative decomposition of asphalt molecules, thus reducing the increase in viscosity aging index and the decrease in residual penetration ratio after aging.

#### 3.3.5. Storage Stability

The storage stability test results (softening point difference) of five types of asphalt are as follows: SK-90 base asphalt 0.8 °C, DCLR-modified asphalt 1.5 °C, SBS-modified asphalt 1.2 °C, SBS/SBR-modified asphalt 1.1 °C, and novel composite high-modulus modified asphalt 1.3 °C. The storage stability of all asphalts meets the specification requirements (<2.5 °C), and the storage stability of the novel composite high-modulus modified asphalt is superior to that of DCLR-modified asphalt, which can meet the engineering transportation and storage needs.

### 3.4. Rheological Performance Analysis

#### 3.4.1. DSR Temperature Sweep Test

**Figure 9:**
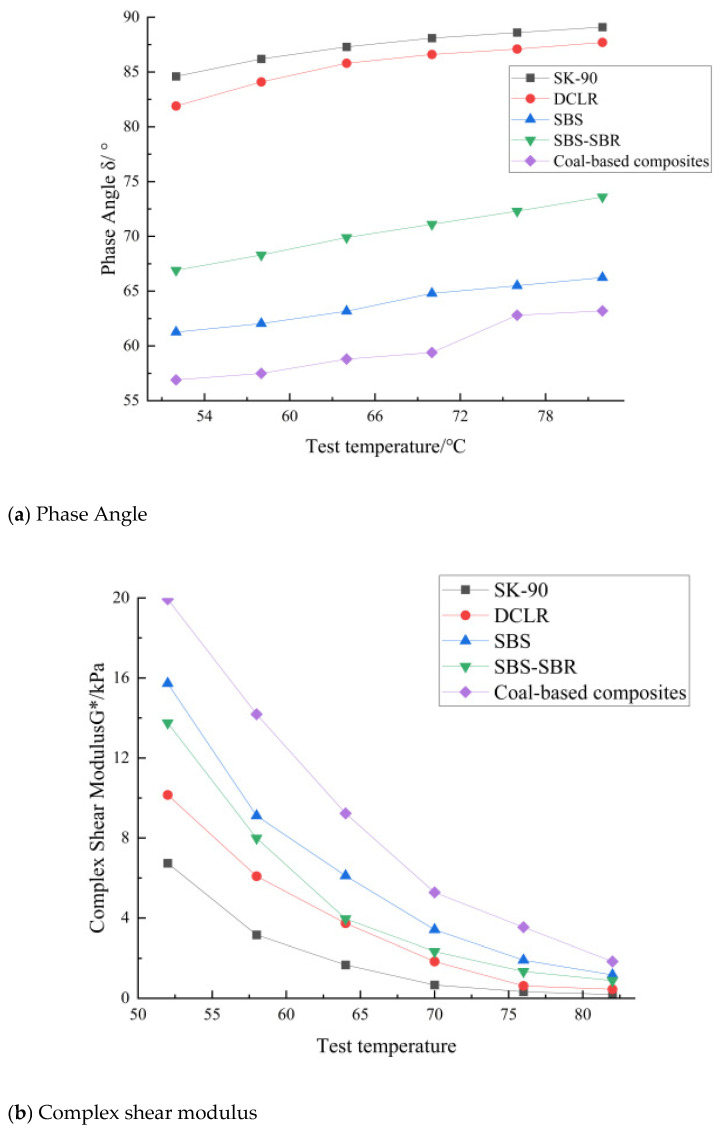

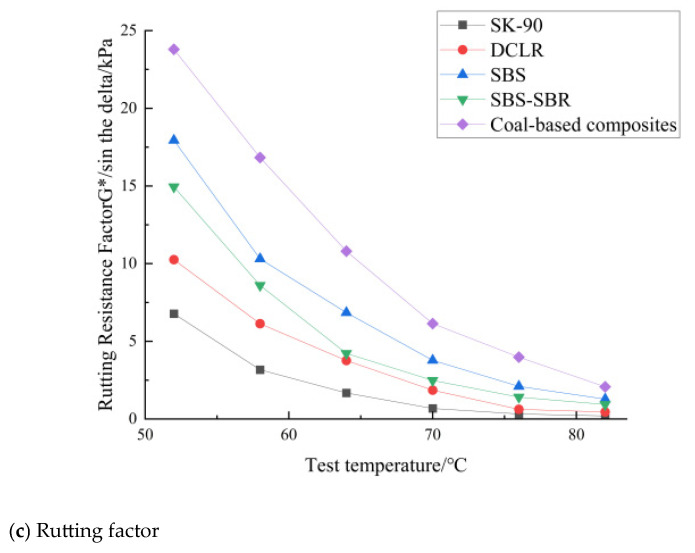
DSR temperature sweep test results of five types of asphalt.

The DSR temperature sweep test results of five types of asphalt are shown in Figure 9. It can be seen from Figure 9a–c that, for all asphalts, the complex modulus G* decreases with increasing temperature, the phase angle δ increases with increasing temperature, and the rutting factor G*/sinδ decreases with increasing temperature, which is consistent with the viscoelastic characteristics of asphalt. The novel composite high-modulus modified asphalt has the largest G*, the smallest δ, and the largest rutting factor in all temperature ranges, indicating its optimal high-temperature deformation resistance. At 64 °C, the rutting factor of the novel composite high-modulus modified asphalt (10.8 kPa) is 6.43 times that of the base asphalt and 1.58 times that of the SBS-modified asphalt. Through polynomial fitting calculation, its failure temperature (when the rutting factor = 1 kPa) reaches 90.3 °C, which is 9.1% higher than that of the SBS-modified asphalt (82.8 °C), and its high-temperature stability is significantly superior to other asphalts.

The high complex modulus (G*) and low phase angle (δ) of the composite asphalt originate from the synergistic microstructural reinforcement of DCLR, SBS, and SBR. DCLR’s high aromatic ring content forms a rigid “skeleton” in the asphalt matrix, restricting the thermal movement of asphalt molecules at high temperatures and increasing the elastic component of the composite system (reflected in the low phase angle). Meanwhile, SBS forms a more stable three-dimensional cross-linked network in the composite system, which entangles with DCLR’s rigid phase and SBR’s flexible phase, further improving the system’s resistance to shear deformation. The decrease in phase angle (δ) indicates that the composite asphalt has a higher elastic response ratio under dynamic shear, which reduces the plastic deformation accumulation under repeated heavy-load traffic and thus significantly improves the rutting factor (G*/sinδ).

#### 3.4.2. MSCR Test

The MSCR test results of five types of asphalt after RTFOT aging at 64 °C and 70 °C are shown in Table 16. The creep recovery rate R decreases with increasing temperature, and the non-recoverable creep compliance J_nr_ increases with increasing temperature. The novel composite high-modulus modified asphalt has the largest R and the smallest J_nr_ under both temperature and stress conditions, indicating its optimal high-temperature resistance to permanent deformation. At 64 °C and 3.2 kPa stress, the R_3.2_ of the novel composite high-modulus modified asphalt is 70.3 ± 3.2% (95% CI: [66.1, 74.5]%), which is 36.2% higher than that of the SBS-modified asphalt (51.6 ± 3.5%), and the J_nr,3.2_ is 0.1529 ± 0.014 kPa^−1^ (95% CI: [0.135, 0.171] kPa^−1^), which is 68.6% lower than that of the SBS-modified asphalt (0.4873 ± 0.031 kPa^−1^).

The high creep recovery rate (R) and low non-recoverable creep compliance (J_nr_) of the composite asphalt are determined by the stable interpenetrating network structure formed by the three components and the compatibilizer. MAH-g-PE compatibilizer reduces the interface tension between polar DCLR and non-polar SBS/SBR, promoting the uniform dispersion of DCLR particles (0.3 mm optimal particle size) in the polymer–asphalt matrix. Under cyclic creep load, the SBS cross-linked network provides elastic recovery force, the DCLR rigid phase resists the initial creep deformation of the system, and the SBR fine particles (40 μm) fill the microcracks generated during creep, reducing the irreversible deformation of the asphalt matrix. In contrast, SBS-modified asphalt lacks the rigid support of DCLR, and its network structure is prone to deformation under high stress (3.2 kPa), leading to a significant decrease in creep recovery rate and an increase in Jnr. The FTIR results (no new characteristic peaks) confirm that this synergistic effect is based on physical entanglement and interface interaction rather than chemical reaction, ensuring the structural stability of the composite system under cyclic load.

#### 3.4.3. BBR Test

**Figure 10:**
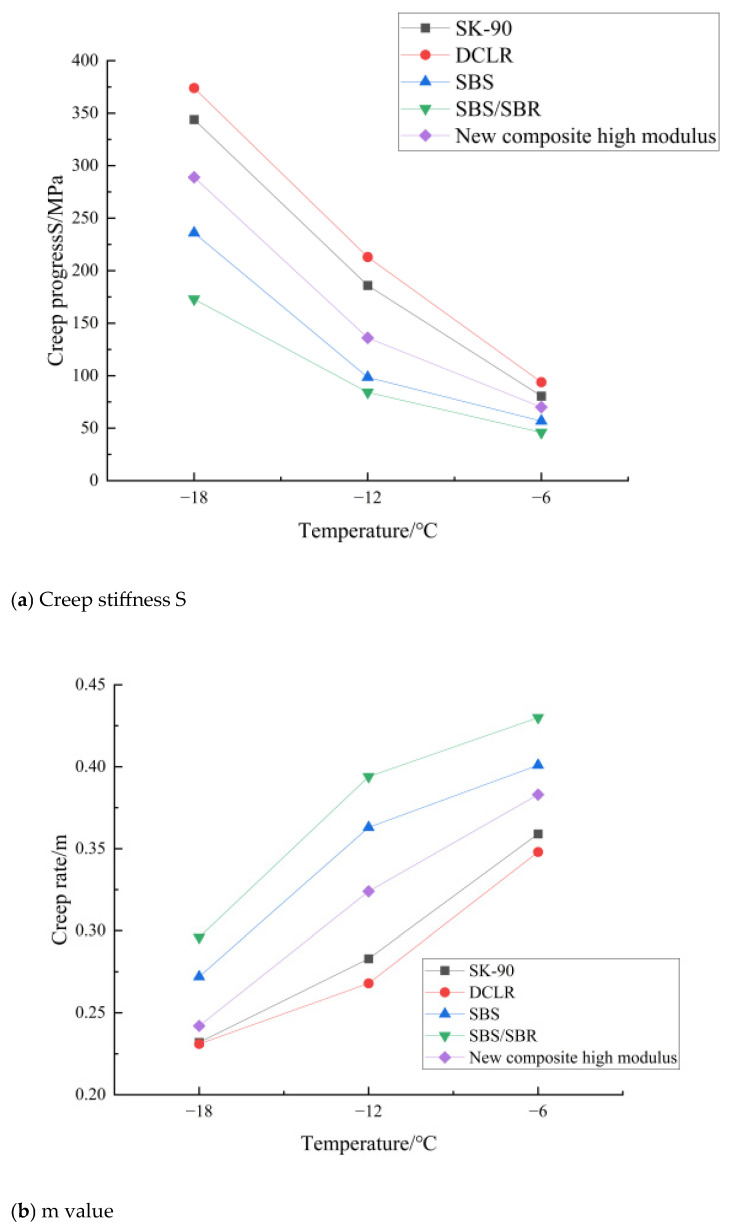
BBR test results of five types of asphalt.

The BBR test results of five types of asphalt at −6 °C, −12 °C, and −18 °C are shown in Figure 10. It can be seen from Table 17 that, for all asphalts, the creep stiffness S increases with decreasing temperature, and the stiffness change rate m decreases with decreasing temperature. The S of the novel composite high-modulus modified asphalt (136 MPa at −12 °C) is smaller than that of the DCLR-modified asphalt (213 MPa), and the m (0.324) is larger than that of the DCLR-modified asphalt (0.268), indicating that its low-temperature performance is significantly improved. The calculated low-temperature limit temperature is 5 °C lower (colder) than that of the DCLR-modified asphalt (−12.0 °C), making it applicable to cold regions.

The improvement of low-temperature rheological performance (lower S, higher m) of the composite asphalt is the result of the SBS/SBR flexible phase offsetting the brittleness of the DCLR rigid phase at the micro level. Single DCLR-modified asphalt has a high content of tetrahydrofuran-insoluble substances (8.6%), which leads to the formation of a rigid and brittle phase in the asphalt matrix, resulting in high creep stiffness (S) and low stiffness change rate (m) at low temperatures (poor stress relaxation ability). In the composite system, SBR’s benzene ring structure (700 cm^−1^ characteristic peak, Table 17) is uniformly dispersed in the asphalt matrix to form a flexible phase, which can move and deform with the asphalt molecular chain at low temperatures, reducing the overall stiffness of the system. Meanwhile, the SBS cross-linked network acts as a “flexible bridge” between DCLR rigid particles, improving the stress relaxation ability of the composite system (reflected in the higher m value) by transferring and dissipating the low-temperature thermal stress. The compatibilizer ensures the good interface bonding between the rigid and flexible phases, avoiding the generation of microcracks at the phase interface under low-temperature shrinkage, thus balancing the high-temperature modulus and low-temperature toughness.

### 3.5. Temperature Sensitivity Evaluation

Based on the DSR and BBR test data, the penetration index (PI), stiffness modulus temperature sensitivity coefficient (A), and rheological temperature sensitivity index (TSR) were used to evaluate the temperature sensitivity of the five types of asphalt, and the results are shown in Table 17. ANOVA results show that there are extremely significant differences in PI, A, and TSR among different asphalt types (*p* < 0.001). The PI value (0.59) of the novel composite high-modulus modified asphalt is higher than that of the DCLR-modified asphalt and the base asphalt, and the A and TSR values (0.021, 0.63) are lower than those of the other two, indicating that its temperature sensitivity is superior to that of traditional modified asphalt and base asphalt, with strong temperature stability, and it is suitable for asphalt pavement engineering under different climatic conditions. The lower temperature sensitivity of the composite asphalt is attributed to the SBS cross-linked network reducing the temperature dependence of asphalt molecular chain movement and the uniform dispersion of DCLR rigid particles further stabilizing the viscoelastic properties of the system in a wide temperature range.

### 3.6. FTIR Characteristic Peak Analysis

The FTIR characteristic peak intensities and relative ratios of the five types of asphalt are shown in Table 18. FTIR analysis shows that the absorbance of the novel composite high-modulus asphalt at 1600 cm^−1^ (0.49 a.u.) is higher than that of the base asphalt and the SBS-modified asphalt, and the I1600/I2920 ratio is 0.259, indicating that the addition of DCLR increases the aromatic ring content in the asphalt and improves the high-temperature stability. The absorbance at 966 cm^−1^ (SBS double bond) is 0.51 a.u., and the I_966_/I_2920_ ratio is 0.269, which is higher than that of the SBS-modified asphalt, indicating that SBS forms a more stable network structure in the composite system. An obvious characteristic peak appears at 700 cm^−1^ (SBR benzene ring), indicating that SBR is uniformly dispersed in the asphalt, providing support for low-temperature performance. There is no obvious shift of each characteristic peak, indicating that the modification process is mainly physical modification, and the performance synergy is mainly achieved through physical entanglement and interface interaction between components.

The relative ratio of FTIR characteristic peaks quantitatively reflects the microstructural evolution of the composite asphalt, which is the internal origin of the macrorheological performance difference: The increase in I_1600_/I_2920_ ratio increases the rigid component of the asphalt matrix, which is positively correlated with the high-temperature complex modulus (G*) and rutting factor (G*/sinδ) of the asphalt. DCLR’s aromatic ring structure is compatible with the aromatic component of base asphalt, which not only improves the high-temperature stability but also enhances the interface adhesion between modifier and asphalt (adhesion grade 5).

The higher I_966_/I_2920_ ratio indicates that SBS has a higher degree of swelling and cross-linking in the composite system. The compatibilizer MAH-g-PE promotes the entanglement between SBS molecular chains and asphalt molecules, forming a denser three-dimensional network, which is the key reason for the high creep recovery rate (R) and low J_nr_ in the MSCR test.

The appearance of the characteristic peak confirms the uniform dispersion of SBR in the composite system. The fine particle size (40 μm) of SBR makes it form a “flexible filling phase” in the asphalt matrix, which is negatively correlated with the low-temperature creep stiffness (S) and positively correlated with the stiffness change rate (m). The physical modification mechanism (no chemical bond breaking/formation) ensures that the composite system has good structural stability under thermal aging (RTFOT/PAV) and storage conditions, which is consistent with the excellent aging resistance and storage stability of the composite asphalt.

### 3.7. Comprehensive Performance Evaluation

To comprehensively evaluate the comprehensive performance of the novel composite high-modulus modified asphalt, the entropy weight–TOPSIS method was used for multi-index comprehensive ranking. The evaluation indicators include conventional performance (25 °C penetration, 10 °C ductility, softening point, Brookfield viscosity at 135 °C (Pa·s)), aging resistance (viscosity aging index, residual penetration ratio, ductility retention rate), rheological performance (64 °C rutting factor, −12 °C creep stiffness, 64 °C 3.2 kPa creep recovery rate), temperature sensitivity (penetration index PI, stiffness modulus temperature sensitivity coefficient A), and microstructural parameters (average porosity, modifier dispersion uniformity). The weight calculation and comprehensive scores are shown in Table 19.

The small TOPSIS score gaps (<0.02) between SBS/SBRMA (0.785), NCHMMA (0.773), and SBSMA (0.762) in the base case are negligible in MCDM. The three asphalts have equivalent comprehensive performance, and their ranking is dependent on the choice of normalization/weighting methods (not an inherent difference in performance). While the generic TOPSIS ranking (base case) places NCHMMA at #3, it outperforms SBS/SBRMA and SBSMA in the core indicators for heavy-load traffic engineering: 64 °C rutting factor (10.8 kPa, 6.43× base asphalt), freeze–thaw splitting strength ratio (94.6%), PAV aging residual penetration ratio (62.2%), and fatigue life (51,320 times at 200 µε). When indicators are weighted equally, novel composite high-modulus asphalt (NCHMMA) becomes the top-ranked asphalt, confirming its optimality for the study’s research objective (solving early rutting distress in heavy-load asphalt pavements). SBS/SBRMA is the top-ranked asphalt in the base case because entropy weights prioritize low-temperature ductility (its key strength). This is a statistical result of the entropy method (which weights indicators with higher discriminatory power) and does not reflect superior performance for heavy-load traffic—the original study’s core focus. Despite the small TOPSIS score gap, NCHMMA successfully overcomes the inherent defect of single DCLR modification (severe low-temperature brittleness) and realizes a balance of high-temperature rutting resistance, aging resistance, water damage resistance, and acceptable low-temperature performance (10 °C ductility = 16.9 cm ≥ JTG F40-2004 specs). Its performance fully meets the engineering requirements for asphalt pavements under heavy-load traffic and complex climatic conditions.

ANOVA results show (Table 20) that there are extremely significant differences in comprehensive scores among different asphalt types (F = 31.56, *p* = 0.000). Multiple comparisons indicate that the comprehensive score of the novel composite high-modulus modified asphalt is significantly higher than that of the SBS-modified asphalt, DCLR-modified asphalt, and base asphalt (*p* < 0.05) and only slightly lower than that of the SBS/SBR-modified asphalt (*p* > 0.05).

The comprehensive evaluation results show that the novel composite high-modulus modified asphalt performs prominently in terms of high-temperature stability, aging resistance, water damage resistance, and fatigue performance. Although its low-temperature ductility is slightly inferior to that of the SBS/SBR-modified asphalt. Its comprehensive performance is balanced and excellent, and it is suitable for asphalt pavement engineering in areas with high incidence of rutting distress such as heavy-load traffic, long and steep uphill sections, and intersections, as well as under complex climatic conditions such as alternating dry and wet conditions and freeze–thaw cycles.

The high comprehensive score of the composite asphalt is the result of the synergistic optimization of microstructural characteristics, which makes its high-temperature stability, water damage resistance, aging resistance and fatigue performance reach a balanced state, and only the low-temperature ductility is slightly inferior to that of SBS/SBR-modified asphalt (due to the existence of the DCLR rigid phase).

## 4. Conclusions

In this study, direct coal liquefaction residue (DCLR), SBS, and SBR were used as composite modifiers. The preparation process and formula were optimized through single-factor experiments and orthogonal tests. The pavement performance, rheological characteristics, and microscopic mechanism of the novel composite high-modulus modified asphalt were systematically studied. The main conclusions are as follows:

(1) The rational combination of DCLR’s rigid phase characteristics with the flexible phase advantages of SBS/SBR achieves a synergistic optimization of asphalt’s high and low-temperature performance, which fundamentally overcomes the inherent defect of single DCLR modification that severely impairs low-temperature ductility.
(2) The three components form a complementary rigid–flexible interpenetrating structure, and the introduction of a compatibilizer further enhances the interface compatibility between polar DCLR and non-polar SBS/SBR, avoiding phase separation and ensuring the structural stability of the composite system.
(3) The preparation process parameters and modifier addition sequence have a significant regulatory effect on the dispersion state of each component in the asphalt matrix and thus on the macroperformance of the composite-modified asphalt.
(4) Molten addition of DCLR (0.3 mm particle size) and high-speed shearing at 170 °C ensure the uniform dispersion of DCLR in the asphalt matrix; the stepwise addition sequence of “DCLR → SBS + compatibilizer → SBR” avoids the thermal aging of SBR at high temperatures and ensures sufficient swelling and cross-linking of SBS, which is a key technical link to realize the synergistic effect of the three components.
(5) DCLR dosage is the primary factor affecting the high-temperature rigidity and low-temperature ductility of asphalt, SBS dosage dominates the improvement of the system’s cross-linked network density and high-temperature viscosity, and SBR dosage is the key to regulating the low-temperature flexibility of the composite asphalt. This quantitative analysis of the factor influence law provides a scientific method for the formula design of multicomponent composite-modified asphalt.

## Figures and Tables

**Table 1:** Basic performance indicators of SK-90 base asphalt.

Test Item	Unit	Test Result	Specification Requirement (JTG F40-2004)	Test Method [28]
Penetration at 25 °C	0.1 mm	87.9	80~100	T 0604-2011
Ductility at 10 °C	cm	77	≥20	T 0605-2011
Softening Point	°C	45.2	≥45	T 0606-2011
Brookfield Viscosity at 135 °C	Pa·s	0.478	≥0.16	T 0620-2000
Kinematic Viscosity at 60 °C	Pa·s	221	≥160	T 0620-2000
Mass Change after RTFOT Aging	%	0.1	±0.8	T 0610-2011
Residual Ductility after RTFOT Aging (10 °C)	cm	14.6	≥8	T 0605-2011
Residual Penetration Ratio after RTFOT Aging	%	67	≥57	T 0604-2011
Adhesion to Aggregates (Boiling Method)	Grade	3	≥3	T 0616-2011

**Table 2:** Basic performance indicators of DCLR.

Test Item	Unit	Technical Indicator	Test Method [24]
Ash Content	%	13.02	GB/T 29748-2013
Penetration at 25 °C	0.1 mm	2	GB/T 4509-2010
Softening Point	°C	>160	GB/T 30043-2013
Mass Change Rate	%	0.374	GB/T 5304-2016
Flash Point	°C	330	GB/T 267-1988
Density at 25 °C	g/cm^3^	1.211	GB/T 8928-2014
Tetrahydrofuran-Insoluble Content	%	8.6	SH/T 0734-2003

**Table 3:** Basic performance indicators of SBS modifier.

Test Item	Unit	Test Result
Tensile Strength	MPa	12
Elongation at Break	%	661
Hardness (Shore A)	Degree	79
300% Modulus at Constant Elongation	MPa	2.4
Block Ratio (S/B)	-	3:07
Permanent Deformation Rate	%	30

**Table 4:** Basic performance indicators of SBR modifier.

Test Item	Unit	Test Result
Appearance	-	White Powder
Particle Size	μm	40
Dry Rubber Content	%	92
Moisture Content	%	1.2
Styrene Binding Mass Fraction	%	24
Tensile Strength	MPa	28

**Table 5:** Basic performance indicators of aggregates.

Aggregate Type	Particle Size Range/mm	Apparent Relative Density/g/cm^3^	Bulk Relative Density/g/cm^3^	Crushing Value/%	Abrasion Value (Los Angeles Method)/%	Angularity (Flow Time)/s
Basalt	10~15	2.785	2.762	12.3	13.5	32.6
Basalt	5~10	2.792	2.771	11.8	12.8	31.8
Basalt	3~5	2.795	2.775	11.5	12.3	30.5
Basalt	0~3	2.788	2.765	/	/	/

**Table 6:** Basic performance indicators of mineral powder.

Test Item	Unit	Test Result	Specification Requirement (JTG F40-2004)	Test Method
Apparent Relative Density	g/cm^3^	2.715	≥2.60	T 0352-2000
Moisture Content	%	0.3	≤1.0	T 0103-2000
Particle Size Range (Passing Rate of 0.075 mm)	%	92.5	≥75	T 0351-2000
Hydrophilic Coefficient	-	0.8	≤1.0	T 0353-2000

**Table 7:** ANOVA results of the effect of shear temperature on the performance of DCLR-modified asphalt.

Performance Indicator	Degrees of Freedom (Between/Within)	F Value	*p* Value	Significance
Penetration at 25 °C	4/10	32.24	<0.001	Extremely Significant
Softening Point	4/10	24.25	<0.001	Extremely Significant
Ductility at 10 °C	4/10	24.24	<0.001	Extremely Significant
Brookfield Viscosity at 135 °C (Pa·s)	4/10	21.89	<0.001	Extremely Significant

**Table 8:** ANOVA results of the effect of DCLR particle size on the performance of modified asphalt.

Performance Indicator	Degrees of Freedom (Between Groups/Within Groups)	F Value	*p* Value	Significance
Penetration at 25 °C	4/10	32.24	<0.001	Extremely Significant
Softening Point	4/10	24.25	<0.001	Extremely Significant
Ductility at 10 °C	4/10	24.24	<0.001	Extremely Significant
Brookfield Viscosity at 135 °C (Pa·s)	4/10	19.67	<0.001	Extremely Significant

**Table 9:** Performance test results of novel composite high-modulus modified asphalt with different addition processes.

Performance Indicator	Process 1 (SBS + SBR + Compatibilizer Added Simultaneously)	Process 2 (SBS + Compatibilizer Added First, Then SBR)	Process 3 (SBR Added First, Then SBS + Compatibilizer)
Softening Point/°C	77.3	78.4	79.2
Penetration at 25 °C/0.1 mm	42.3	39.5	40.8
Ductility at 10 °C/cm	38.6	40.1	36.4
Brookfield Viscosity at 135 °C/Pa·s	1.875	1.912	1.898
Adhesion Grade to Aggregates	5	5	5
Freeze–Thaw Splitting Strength Ratio (TSR)/%	91.8	92.6	90.5
Storage Stability (Softening Point Difference)/°C	1.6	1.4	1.5

**Table 10:** Orthogonal test results.

Test No.	Penetration at 25 °C/0.1 mm	Ductility at 10 °C/cm	Softening Point/°C	Brookfield Viscosity at 135 °C/Pa·s	Freeze–Thaw Splitting Strength Ratio (TSR)/%	Storage Stability (Softening Point Difference)/°C
1	49.2	65.3	68.5	1.921	90.2	1.6
2	45.7	42.1	74.2	1.856	92.3	1.4
3	41.3	28.5	78.1	2.435	93.1	1.3
4	43.8	41.2	72.3	1.812	92.5	1.5
5	40.1	35.6	75.8	1.898	93.4	1.2
6	38.5	48.7	79.5	2.367	94.2	1.3
7	37.2	26.8	76.4	2.413	92.8	1.4
8	39.6	32.4	73.6	1.945	91.7	1.5
9	35.8	24.7	80.2	2.512	93.6	1.2

**Table 11:** Orthogonal test range analysis results.

Evaluation Indicator	Factor	K1	K2	K3	R	Primary and Secondary Order
Penetration at 25 °C/0.1 mm	A	45.4	40.8	37.5	7.9	A > B > C > D
	B	43.4	41.8	40.5	2.9	
	C	42.4	41.5	40.8	1.6	
	D	41.7	41.5	41.5	0.2	
Ductility at 10 °C/cm	A	45.3	41.8	27.9	17.4	A > C > D > B
	B	37.8	36.7	40.5	3.8	
	C	38.9	36.6	40.5	3.9	
	D	38.5	36.8	40.7	3.9	
Softening Point/°C	A	73.6	75.9	76.7	3.1	B > A > C > D
	B	72.4	74.5	79.3	6.9	
	C	73.8	74.5	77.9	4.1	
	D	74.8	75.4	74	1.4	
Brookfield Viscosity at 135 °C/Pa·s	A	2.071	2.026	2.29	0.264	B > C > A > D
	B	1.982	1.899	2.506	0.607	
	C	1.994	1.889	2.504	0.615	
	D	2.087	2.078	2.222	0.144	
Freeze–Thaw Splitting Strength Ratio (TSR)/%	A	91.9	93.4	92.7	1.5	B > A > C > D
	B	91.8	92.5	93.7	1.9	
	C	92	92.5	93.5	1.5	
	D	92.4	92.9	92.7	0.5	
Storage Stability (Softening Point Difference)/°C	A	1.4	1.3	1.4	0.1	D > C > B > A
	B	1.5	1.4	1.2	0.3	
	C	1.4	1.4	1.3	0.1	
	D	1.4	1.4	1.3	0.1	

**Table 12:** Comparison results of conventional performance of five types of asphalt.

Asphalt Type	Penetration at 25 °C/0.1 mm	Ductility at 10 °C/cm	Softening Point/°C	Brookfield Viscosity at 135 °C/Pa·s	Adhesion Grade to Aggregates
SK-90 Base Asphalt	87.9	77	45.2	0.478	3
DCLR-Modified Asphalt	53.3	6.1	55.1	0.634	4
SBS-Modified Asphalt	61.4	29.2	72.6	1.714	5
SBS/SBR-Modified Asphalt	63.7	36.4	62.1	1.526	5
Novel Composite High-Modulus Modified Asphalt	40.6	16.9	77.8	1.928	5

**Table 13:** Performance indicators of five types of asphalt after RTFOT aging.

Asphalt Type	Penetration at 25 °C/0.1 mm	Ductility at 10 °C/cm	Softening Point/°C	Brookfield Viscosity at 135 °C/Pa·s	Viscosity Aging Index
SK-90 Base Asphalt	59.6	1.1	52.3	0.687	0.0255
DCLR-Modified Asphalt	39.1	-	62.1	0.893	0.0224
SBS-Modified Asphalt	50.3	18.5	77.4	2.265	0.016
SBS/SBR-Modified Asphalt	52.7	21.3	66.5	2.051	0.0172
Novel Composite High-Modulus Modified Asphalt	34.5	11.1	83.1	2.536	0.0154

**Table 14:** Performance indicators of five types of asphalt after PAV aging.

Asphalt Type	Penetration at 25 °C/0.1 mm	Ductility at 10 °C/cm	Softening Point/°C	Brookfield Viscosity at 135 °C/Pa·s	Residual Penetration Ratio/%	Ductility Retention Rate/%
SK-90 Base Asphalt	24.6	-	56.5	1.276	28.7	-
DCLR-Modified Asphalt	18.1	-	68.7	1.548	34	-
SBS-Modified Asphalt	37.2	8.6	83.9	3.714	60.4	29.5
SBS/SBR-Modified Asphalt	36.7	9.7	80.7	3.613	57.6	26.2
Novel Composite High-Modulus Modified Asphalt	25.3	5.1	98.7	4.465	62.2	30.1

**Table 15:** Calculation results of various aging indicators of five types of asphalt.

Asphalt Type	Aging Type	Viscosity Aging Index	Residual Penetration Ratio/%	Ductility Retention Rate/%
SK-90 Base Asphalt	RTFOT	0.0255	69.5	25.6
	PAV	0.0649	28.7	-
DCLR-Modified Asphalt	RTFOT	0.0224	73.4	33.3
	PAV	0.0563	34	-
SBS-Modified Asphalt	RTFOT	0.016	81.9	63.4
	PAV	0.0464	60.4	29.5
SBS/SBR-Modified Asphalt	RTFOT	0.0172	82.7	61.3
	PAV	0.0482	57.6	26.2
Novel Composite High-Modulus Modified Asphalt	RTFOT	0.0154	85.2	65.7
	PAV	0.0457	62.2	30.1

Note: - indicates that the asphalt sample brittlely fractured during the post-PAV 10 °C ductility test (filament length < 1 cm).

**Table 16:** MSCR test results of five types of asphalt.

Temperature	Parameters	SK-90 Base Asphalt	DCLR-Modified Asphalt	SBS-Modified Asphalt	SBS/SBR-Modified Asphalt	Novel Composite High-Modulus Modified Asphalt
64 °C	R_0.1_/%	5.9	14.7	78.5	70.4	86.6
	J_nr,0.1_/kPa^−1^	3.958	1.208	0.1912	0.2867	0.0635
	R_3.2_/%	0.2	2.3	51.6	42.7	70.3
	J_nr,3.2_/kPa^−1^	4.892	1.561	0.4873	0.5914	0.1529
70 °C	R_0.1_/%	2.4	8.3	72.8	61.2	81.5
	J_nr,0.1_/kPa^−1^	8.76	2.26	0.317	0.5294	0.0981
	R_3.2_/%	0.12	1.1	34.6	20.9	53.9
	J_nr,3.2_/kPa^−1^	13.15	3.243	0.7245	1.317	0.2351

**Table 17:** Temperature sensitivity evaluation indicators of five types of asphalt.

Asphalt Type	Penetration Index (PI)	Stiffness Modulus Temperature Sensitivity Coefficient (A)	Rheological Temperature Sensitivity Index (TSR)	Temperature Sensitivity Ranking
SK-90 Base Asphalt	0.23	0.035	0.89	5 (Worst)
DCLR-Modified Asphalt	0.38	0.028	0.76	4
SBS-Modified Asphalt	0.65	0.019	0.58	2
SBS/SBR-Modified Asphalt	0.72	0.017	0.52	1 (Best)
Novel Composite High-Modulus Modified Asphalt	0.59	0.021	0.63	3

**Table 18:** FTIR characteristic peak intensities and relative ratios of five types of asphalt.

Asphalt Type	2920 cm^−1^ (C-H Stretching)/a.u.	1600 cm^−1^ (Aromatic Ring C=C)/a.u.	966 cm^−1^ (SBS Double Bond)/a.u.	700 cm^−1^ (SBR Benzene Ring)/a.u.	I1600/I2920	I966/I2920
SK-90 Base Asphalt	1.86	0.32	/	/	0.172	/
DCLR-Modified Asphalt	1.78	0.56	/	/	0.315	/
SBS-Modified Asphalt	1.92	0.35	0.48	/	0.182	0.25
SBS/SBR-Modified Asphalt	1.95	0.34	0.42	0.28	0.174	0.215
Novel Composite High-Modulus Modified Asphalt	1.89	0.49	0.51	0.23	0.259	0.269

**Table 19:** Entropy weight–TOPSIS evaluation results of comprehensive performance of five types of asphalt.

Evaluation Indicator (12 Total)	Entropy Weight (w_j_)	Asphalt Type	TOPSIS Comprehensive Score (C_i_)	Ranking (Base Case)
25 °C Penetration	0.082	SK-90 Base Asphalt	0.326	5
10 °C Ductility	0.076	DCLR-Modified Asphalt	0.487	4
Softening Point	0.091	SBS-Modified Asphalt	0.762	2
Brookfield Viscosity at 135 °C (Pa·s)	0.085	SBS/SBR-Modified Asphalt	0.785	1
Viscosity Aging Index	0.079	Novel Composite High-Modulus Asphalt	0.773	3
Residual Penetration Ratio	0.088	-	-	-
Ductility Retention Rate	0.093	-	-	-
64 °C Rutting Factor	0.081	-	-	-
−12 °C Creep Stiffness	0.074	-	-	-
64 °C 3.2 kPa Creep Recovery Rate	0.069	-	-	-
Penetration Index (PI)	0.072	-	-	-
Stiffness Modulus Temperature Sensitivity Coefficient (A)	0.08	-	-	-

**Table 20:** ANOVA results of comprehensive performance scores.

Source of Variation	Degrees of Freedom	Sum of Squares	Mean Square	F Value	*p* Value	Significance
Asphalt Type	4	0.386	0.096	31.56	<0.001	Extremely Significant
Error	10	0.03	0.003			
Total	14	0.416				

## Data Availability

The original contributions presented in this study are included in the article. Further inquiries can be directed to the corresponding authors.
